# Occlusal disharmony-induced stress causes osteopenia of the lumbar vertebrae and long bones in mice

**DOI:** 10.1038/s41598-017-18037-y

**Published:** 2018-01-09

**Authors:** Yasuhiro Shimizu, Masud Khan, Genki Kato, Kazuhiro Aoki, Takashi Ono

**Affiliations:** 1 Department of Orthodontic Science, Graduate School of Medical and Dental Sciences, Tokyo Medical and Dental University (TMDU), 1-5-45 Yushima, Bunkyo-ku, Tokyo 113-8549 Japan; 2 Department of Basic Oral Health Engineering, Graduate School of Medical and Dental Sciences, Tokyo Medical and Dental University (TMDU), 1-5-45 Yushima, Bunkyo-ku, Tokyo 113-8549 Japan; 3 JSPS International Research Fellows, 1-5-45, Yushima, Bunkyo-ku, Tokyo, 113-8549 Japan; 4 Department of Bio-Matrix (Pharmacology), Graduate School of Medical and Dental Sciences, Tokyo Medical and Dental University (TMDU), 1-5-45 Yushima, Bunkyo-ku, Tokyo 113-8549 Japan

## Abstract

Excessive exposure to glucocorticoids causes osteoporosis in children and adults. Occlusal disharmony is known to induce an increase in serum corticosteroid levels in murine models, but the influence of occlusal disharmony-induced stress on the bone mass during the growth period has not yet been clarified. The purpose of this study was to investigate whether occlusal disharmony-induced stress decreases bone mass. Five-week-old C57BL/6J male mice were used. A 0.5-mm increase in the vertical height of occlusion was used to induce occlusal disharmony for a period of 7 days. Serum corticosterone levels were significantly higher on post-induction day 7, with radiological evidence of osteopenia of the third lumbar vertebra and long bones of the hind limbs. Osteopenia was associated with a reduction of the mechanical properties of the tibia and femur, with significant suppression of bone formation parameters and an increase in bone resorption parameters, as evaluated by bone histomorphometric analysis of the tibial/femur metaphysis. Our findings at the level of bones were supported by our assessment of serum markers of systemic metabolism. Therefore, occlusal disharmony-induced stress may lead to osteopenia and reduce the mechanical strength of bone through an increase in serum glucocorticoid levels in mice.

## Introduction

Occlusal disharmony frequently occurs during growth due to mixed dentition, as the primary dentition is changing to permanent dentition. Several lines of evidence indicate that occlusal disharmony increases serum corticosteroid levels in young animals. As the serum corticosteroid level is considered to be a marker of ‘stress’ in rodents, it is plausible that occlusal disharmony would cause stress^1–4^. The high frequency of bruxism (teeth grinding) during the period of growth further suggests the possible existence of occlusal disharmony-induced stress in childhood^5–7^. By contrast, it is well known that excessive exposure to glucocorticoids causes osteopenia^8^. As an example, the use of steroid medications in children for the treatment of immune-related diseases, such as asthma and skin allergies, commonly results in bone-related side effects, including fractures and slowed bone growth^9^. Given the significant effects of stress-induced hormone on bone metabolism^10^, evaluation of the effect of occlusal disharmony-induced stress on skeletal tissue is warranted.

Our aim in this study was to use a mouse model of occlusal disharmony to evaluate the effects of occlusal disharmony-induced stress on bone mass and bone mineral density, as well as on the mechanical properties of the long bones and lumbar vertebrae, which are expected to be differentially affected by mechanical loading^11–13^.

## Results

### Increase in serum corticosterone level with occlusal disharmony

**Figure 1:**
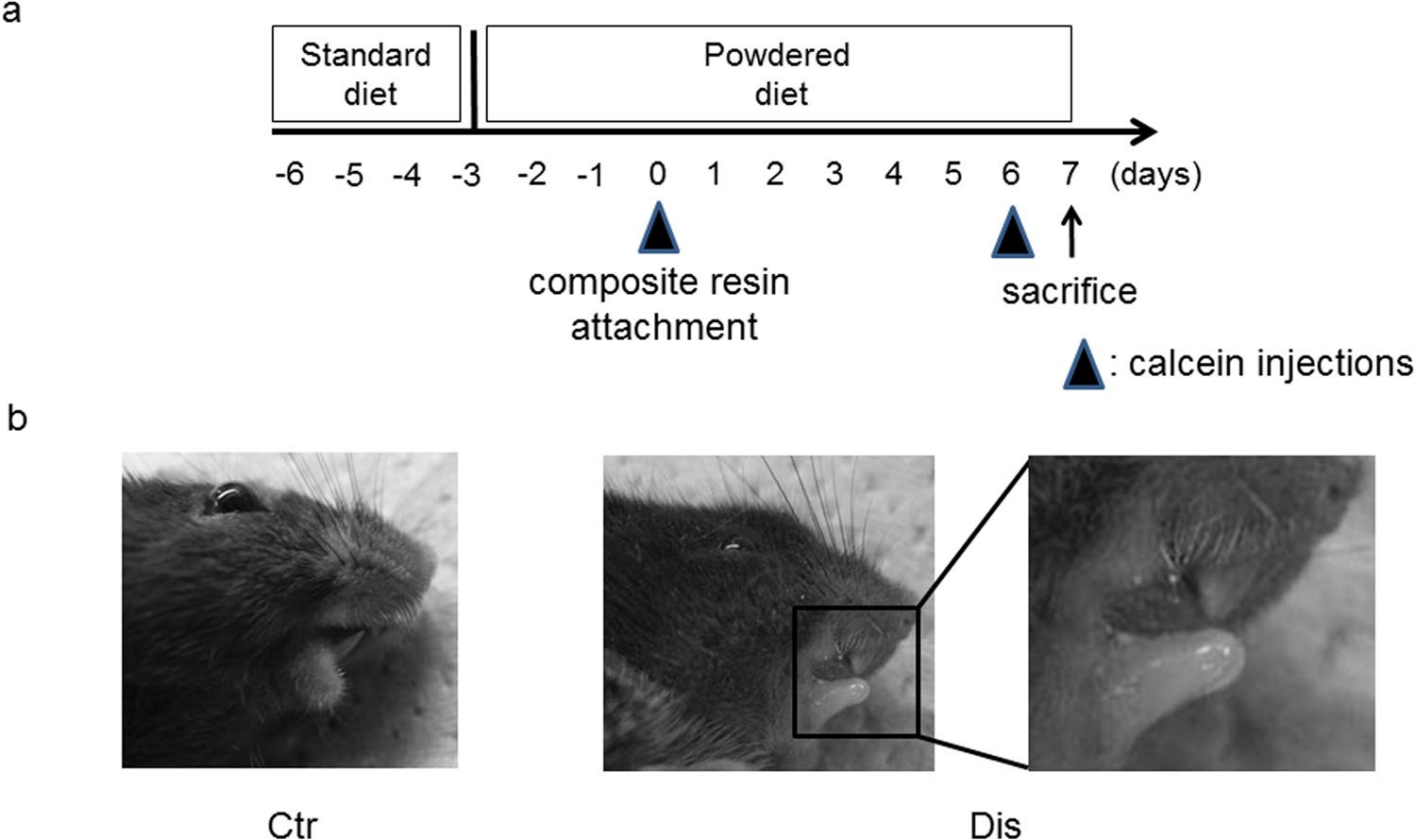
The occlusal disharmony model, showing (**a**) the experimental protocol and (**b**) the occlusal height adjustment, using a composite resin to increase the occlusal height. The height of the lower incisors was adjusted by 0.5 mm in the vertical direction. Ctr, control group; Dis, occlusal disharmony group.

**Figure 2:**
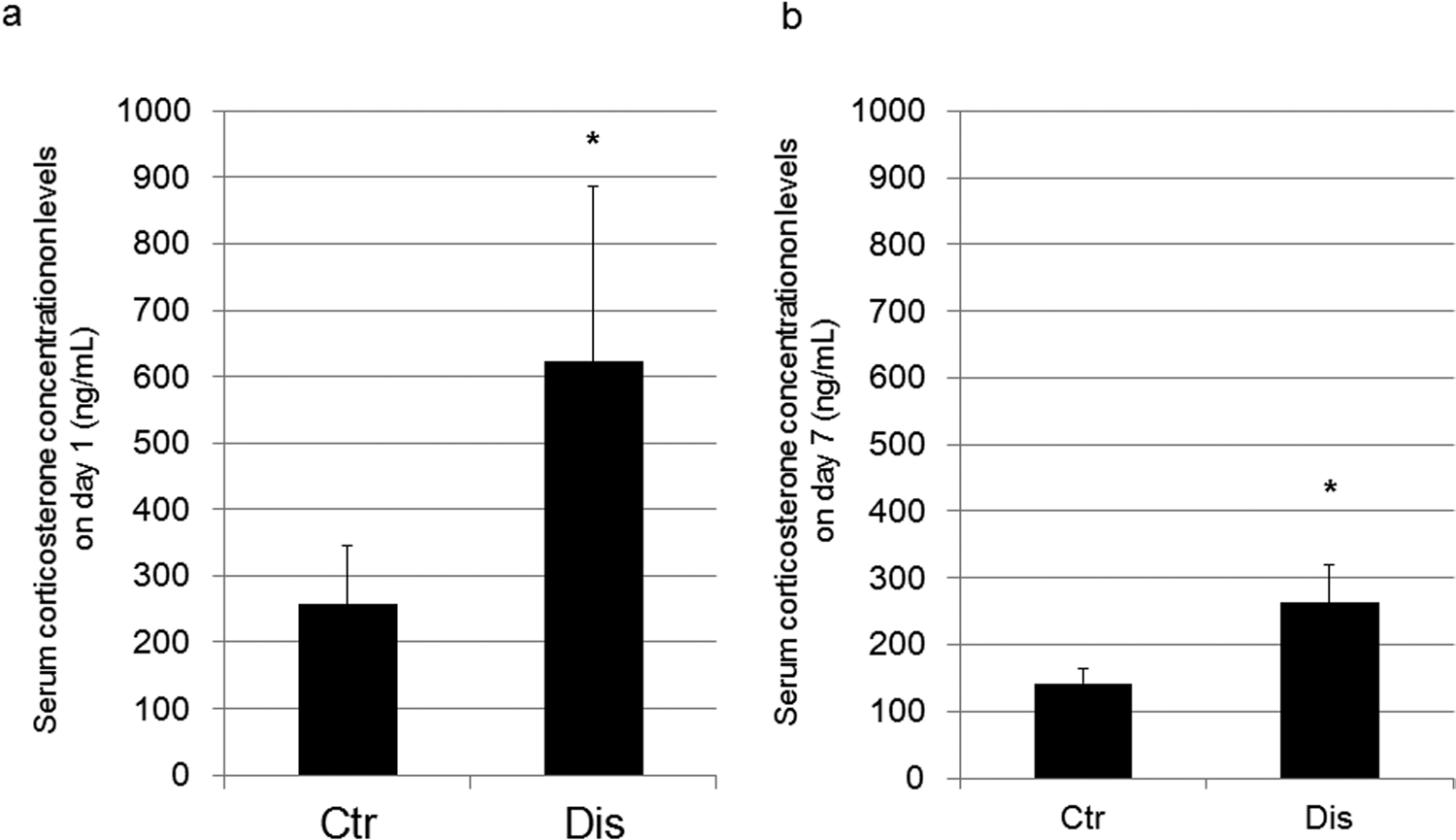
Measurements of serum corticosterone levels (**a**) on day 1 after induction of occlusal disharmony, and (**b**) on post-induction day 7. Values are expressed as the mean ± SD; Ctr, control group; Dis, occlusal disharmony group; *p < 0.05 versus the control group (Ctr).

Serum corticosterone levels were measured at baseline and after the induction of occlusal disharmony (Fig. 1). Serum corticosterone levels were significantly higher in the disharmony than in the control group on post-induction days 1, 3 and 7 (Fig. 2, Supplementary Fig. S1).

### Osteopenia associated with occlusal disharmony

**Figure 3:**
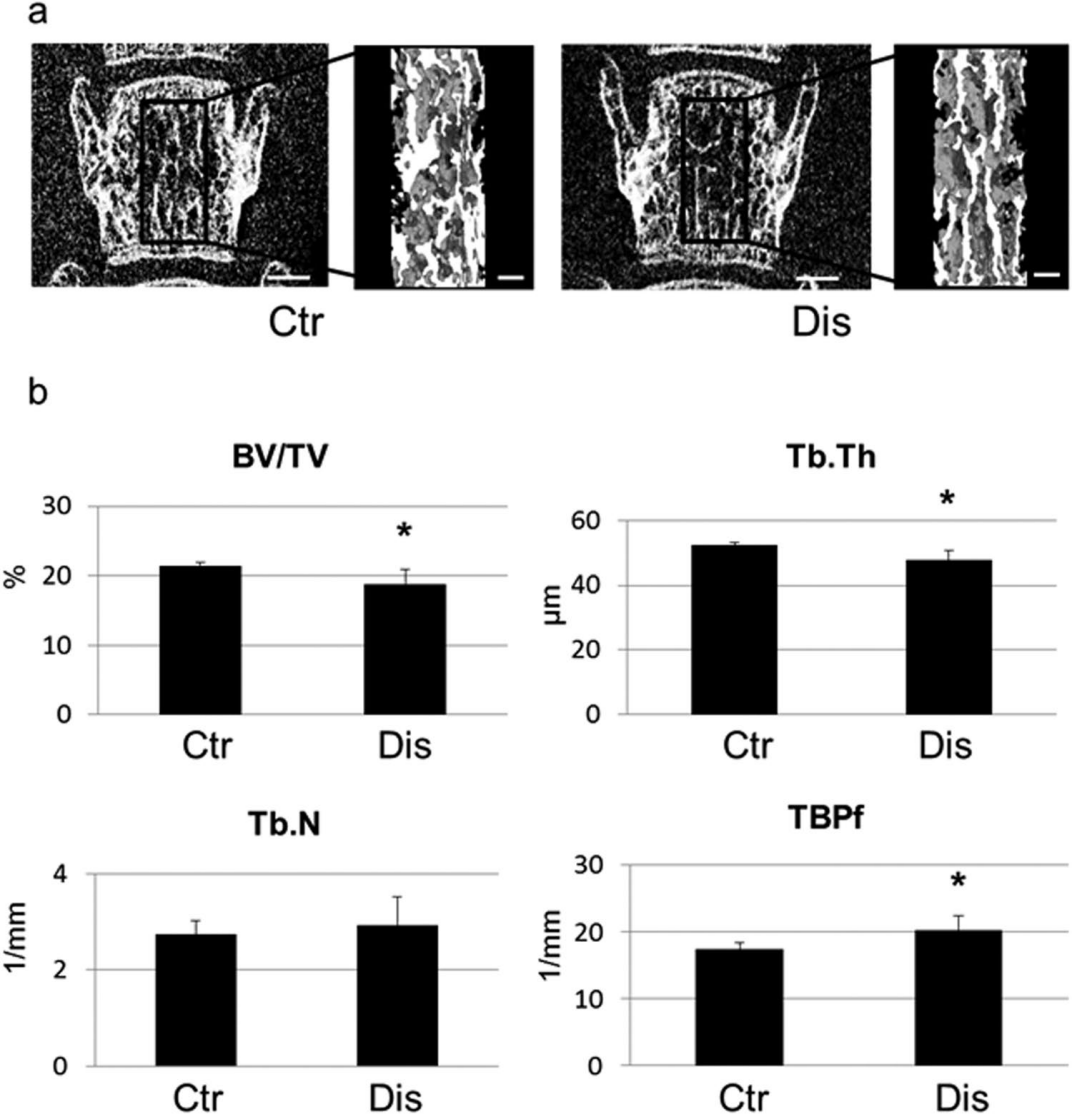
Representative three-dimensional micro-CT images of the structural changes of the trabecular bone of the third lumbar vertebra is shown (**a**); the bars represent 600 μm and 200 μm in low and high magnified images, respectively. The bone structural parameters are shown in (**b**): the percentage of bone volume per tissue volume (BV/TV, %), trabecular thickness (Tb.Th, μm), trabecular number (Tb.N, 1/mm), and trabecular bone pattern factor (TBPf, 1/mm). Values are expressed as the mean ± SD; Ctr, control group; Dis, occlusal disharmony group; *p < 0.05 versus the control group (Ctr).

Micro-computed tomography (CT) analysis provided evidence of osteopenia of the third lumbar vertebra in the disharmony group, which was associated with a significant decrease in structural parameters (bone volume and trabecular thickness), as well as an increase in trabecular bone pattern factor (TBPf, 1/mm), which is indicative of a disconnected trabecular structure (Fig. 3). The association between occlusal disharmony and osteopenia was confirmed at the femoral neck, an area known to be influenced by excessive exposure to glucocorticoids^8^.

### Decrease in bone mineral density and the mechanical parameters of long bones with occlusal disharmony

**Figure 4:**
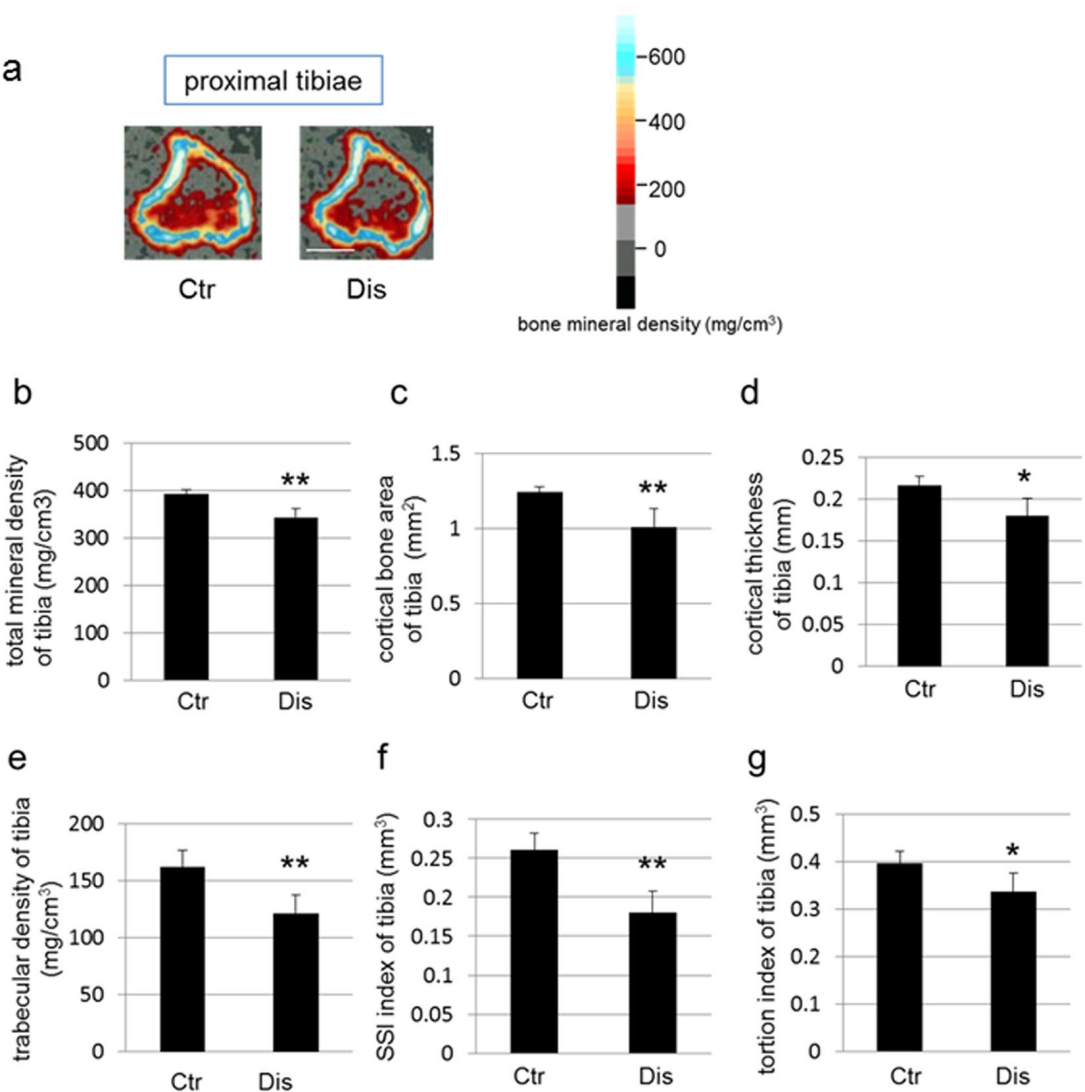
A representative pQCT image of the proximal tibia for quantitative analyses (bar, 1 mm) is shown in (**a**). The colour calibration for bone mineral density is shown in the right bar. The reduction in maximum bone mineral density with occlusal disharmony is indicated. Other measured variables of bone structure are shown as follows: total mineral density of the proximal tibia (**b**); total mineral density of tibia (**c**); cortical bone area of the proximal tibia (**d**); cortical thickness of the proximal tibia (**e**); trabecular density of the proximal tibia (**f**); strength strain index (SSI) of the proximal tibia (**g**); and torsion index of the proximal tibia. Values are expressed as the mean ± SD; Ctr, control group; Dis, occlusal disharmony group; *p < 0.05; **p < 0.01 versus the control group (Ctr).

Effects of occlusal disharmony on the mechanical properties of long bones, and specifically on cortical and trabecular bone, were evaluated by pQCT analysis. Total bone mineral density, the trabecular index of bone mineral density and cortex-related indices, namely cortical bone area and cortical thickness, were significantly suppressed in the tibial metaphysis of mice in the disharmony group, compared to those in the control group (Fig. 4a–e). The mechanical strength indices (SSI and torsion index) of the tibial metaphysis were also significantly lower in the disharmony group than in the control group (Fig. 4f,g). Similarly, the total bone mineral density, the trabecular related index, the cortex-related indices, and the mechanical strength parameters were also significantly lower in the distal femur of mice in the disharmony group than those in the control group. Lower total bone mineral density and cortex-related indices were also observed in the region of the femur (Supplementary Fig. S2).

### Decreased bone formation parameters in the secondary spongiosa of the tibial metaphysis with occlusal disharmony

**Figure 5:**
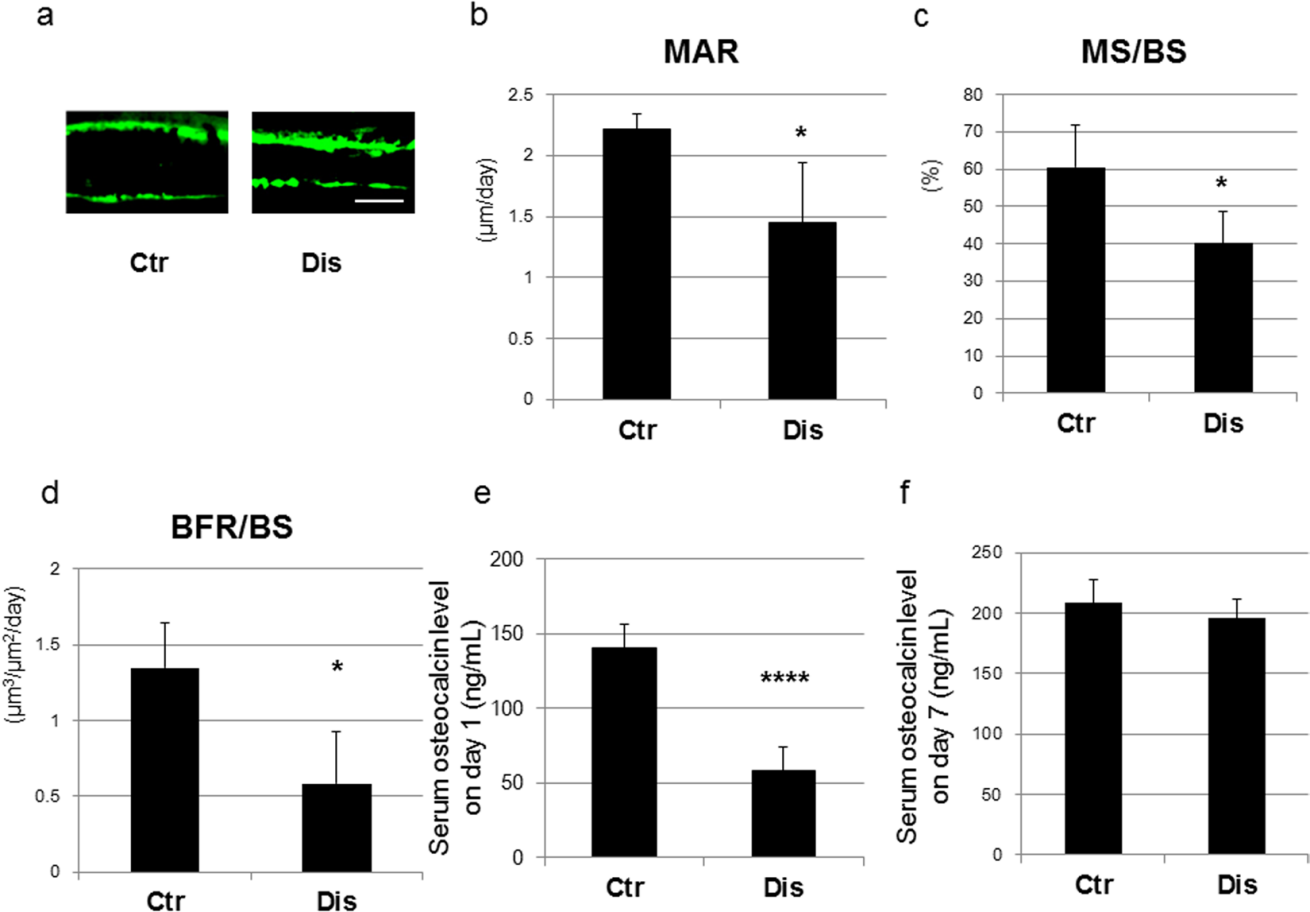
Bone formation parameters, with calcein injections administered at the time of induction of occlusal disharmony (day 0) and on post-induction (day 6). Measured parameters were as follows: (**a**) magnified images of calcein double labelling of trabecular bone (bar, 10 μm); (**b**) mineral apposition rate (MAR); (**c**) mineralizing surface per bone surface (MS/BS); (**d**) bone formation rate (BFR); (**e**) serum osteocalcin level on day 1; and (**f**) serum osteocalcin level on day 7. Values are expressed as the mean ± SD; Ctr, control group; Dis, occlusal disharmony group; *p < 0.05 versus the control group (Ctr); ****p < 0.0001 versus the control group (Ctr).

Calcein injections were administered on the day of induction and on post-induction day 6 to evaluate effects of occlusal disharmony on bone formation activity. Evaluation of un-decalcified thin sections under fluorescent microscopy revealed a decrease in the distance between the calcein double labels in disharmony group, compared to the control group (Fig. 5a). A bone histomorphometric study was performed to confirm these histological observations. The mineral apposition rate (MAR), which is indicative of the bone formation capacity of osteoblasts, was significantly decreased in the disharmony group, compared to the control group (Fig. 5b), along with a smaller mineralizing surface per bone surface (MS/BS; Fig. 5c). Consequently, changes in the bone formation rate (BFR), which reflects the total amount of bone formation in a day, were in the same direction as those in MAR and MS/BS (BFR is calculated as MS/BS × MAR), being significantly lower in the occlusal disharmony than in the control group (Fig. 5d). Serum osteocalcin levels were measured to clarify effects of occlusal disharmony on systemic bone formation activity, with significant decreases in serum levels identified on post-induction day 1 (Fig. 5e).

**Figure 6:**
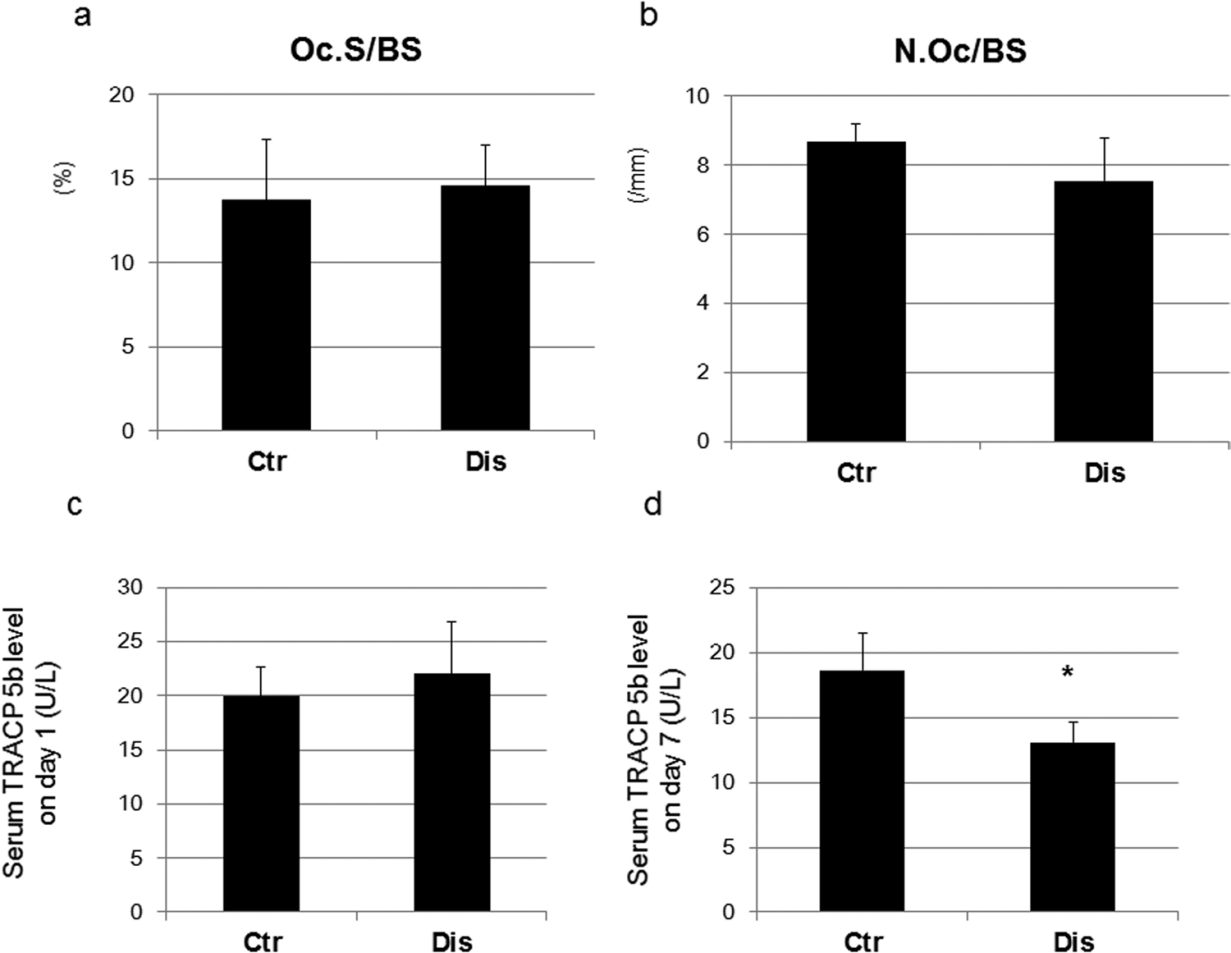
Bone resorption parameters showing: (**a**) the osteoclast surface (Oc.S/BS); (**b**) the number of osteoclasts (N.Oc/BV); (**c**) serum TRACP 5b level on day 1.; and (**d**) serum TRACP 5b level on day 7. Values are expressed as the mean ± SD; Ctr, control group; Dis, occlusal disharmony group; *p < 0.05 versus the control group (Ctr).

**Figure 7:**
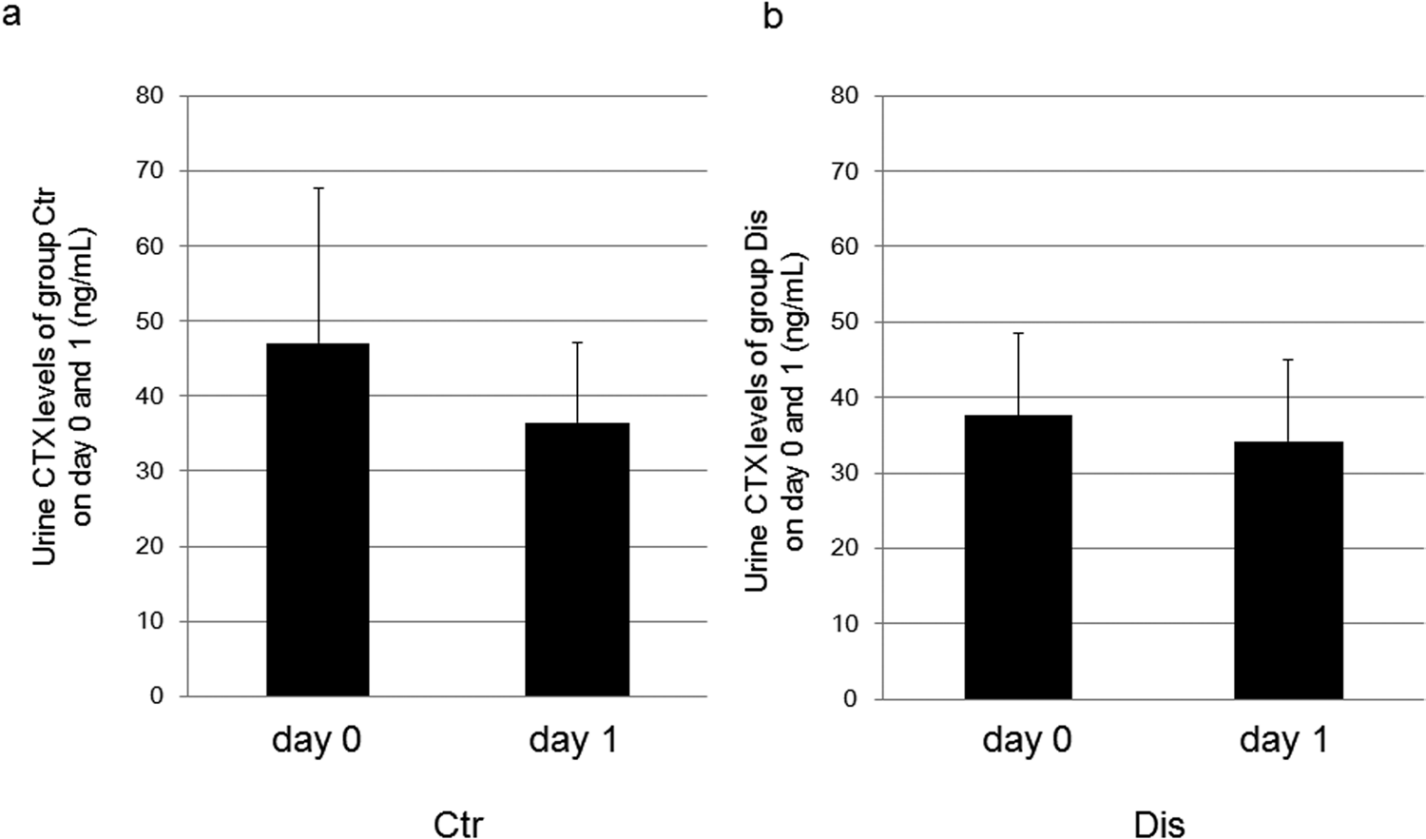
(**a**) Urine CTX levels of the control (Ctr) group on days 0 and 1. (**b**) Urine CTX levels of group Dis on days 0 and 1. Values are expressed as the mean ± SD; Ctr, control group; Dis, occlusal disharmony group.

The osteoclast surface (Oc.S/BS) and the number of osteoclasts (N.Oc/BV) were comparable between the disharmony and control groups (Fig. 6a,b). Serum TRACP 5b level, which is indicative of bone resorption activity, was not modified on post-induction day 1, but did decrease significantly in the disharmony group on post-induction day 7 (Fig. 6c and d). Urine CTX levels were comparable between the disharmony and control groups on day 0 and on post-induction day 1 (Fig. 7a and b).

### High-turnover bone remodelling in the middle of the observation period in the occlusal disharmony group

Histopathological examination of the cortical bone of the femur showed an increase in bone formation in the experimental group on day 7 (Supplementary Figs S3 and S4a). Moreover, the serum level of TRACP 5b, obtained in the middle of the period of observation, was indicative of a significant increase in bone resorption activity in the disharmony group on post-induction day 5 (Supplementary Fig. S4b). These results are suggestive of a high turnover rate of bone remodelling in the middle of the observation period.

### Increased osteocyte apoptosis in the cortical bone area in the occlusal disharmony group

Histological examination of the cortical bone area of the femur, using nuclear staining with DNA-specific fluorescent Hoechst 33258 on post-induction day 3, revealed an increase in chromatin condensation in osteocytes in the occlusal disharmony group (Supplementary Fig. S4b).

## Discussion

Our aim was to evaluate potential effects of occlusal disharmony during growth, using an animal model that has previously been used in research. Our research was motivated by the knowledge of the negative impact of high serum glucocorticoid levels, as well as inadequate nutrition and low physical activity, on the development of peak bone mass in early life^8,9,14–17^. We provide evidence of an increase in serum corticosterone levels with occlusal disharmony, with secondary effects on the bone, which are consistent with previously reported findings^1–4^. Faecal corticosterone levels were not influenced by the modification in diet, namely from pellet to powder, in either the disharmony or the control group. Moreover, as all other experimental conditions, except the use of the composite resin, were comparable between the disharmony and control groups, the measured increase in serum corticosterone levels can only be explained by the induction of occlusal disharmony. The increase in serum levels of corticosterone in the disharmony group exceeded the 200 ng/ml threshold considered to reflect ‘stress’ in rodents (Fig. 2)^3,18^.

Following the induction of occlusal disharmony, body weight decreased for several days, with animals subsequently recovering to a level where there was no significant difference in body weight between animals in the disharmony and control group, as has been previously reported. Certainly, the acute decrease in food consumption following induction of occlusal disharmony might have influenced the bone loss in the experimental group. Previous studies, using an OVX model, have reported an initial increase in bone resorption markers immediately following a decrease in calcium intake, which was followed by an increase in the level of bone formation markers^19^. However, we did not identify this trend in our model, with no increase in either bone formation or resorption markers on post-induction day 1, indicating that the increase in glucocorticoid levels was the main cause of bone loss.

Serum levels of corticosterone were increased after induction of occlusal disharmony, with a 2.4-fold increase in the disharmony group compared with the control group on post-induction day 1, a 2.3-fold increase on post-induction day 3 and a 1.5-fold increase on post-induction day 7 (Fig. 2, Supplementary Fig. S1). It is possible that serum levels of corticosterone would continue to decrease over time in the disharmony group, as animals adapt to the stress of occlusal disharmony. However, we have confirmed the induction of significantly higher serum corticosterone levels in our model during the experimental period, with the corticosteroid level in the experimental group significantly decreasing on day 7, compared to initial stage (day 1 and 3) of occlusal disharmony, this difference confirmed by multiple intergroup comparisons using Fisher’s protected least significant difference (PLSD) as a post hoc test (Supplementary Fig. S5). We also observed that the increase in bone resorption marker identified on day 5 in the occlusal disharmony group was no longer evident on day 7, a result which might be comparable to using a GR antagonist treatment (Supplementary Fig. S8). Further research is warranted, including the use of GR antagonist treatment, to clarify the causal effect of increased serum corticosteroid levels on bone in the occlusal disharmony group.

Previous studies have reported that, following an initial adaptation to stress, occlusal disharmony increases the response of the hypothalamic–pituitary–adrenal (HPA) axis to subsequent new stress. Therefore, eliminating stress induced by malocclusion during the period of growth could be important to optimize bone growth and the development of peak bone mass, thereby, lowering the risk for future osteoporotic fractures^3,8,9,14–17^.

One of the factors that induces bone loss is a reduction of locomotor activity^11^. Certainly, previous studies have reported a decrease in locomotor activity associated with an increase in stress^12^. However, locomotor activity is not considered to be a significant factor of vertebral bone loss in murine models^13^. Moreover, in our study, bone loss in the third lumbar vertebra was comparable to the loss in the long bones of the hind limbs, indicative that the increase in glucocorticoid levels was the main factor inducing bone loss in our model.

Occlusal disharmony was induced by increasing occlusal height. The resultant increase in serum corticosterone level led to a reduction in both cortical and trabecular bone mass of the long bones of the hind limb. In our previous models of osteopenia, including mice fed with a low-calcium diet, we reported a significant reduction in trabecular bone within the first week, with the cortical bone being less affected by the interventions^20,21^. In comparison to these previous models of osteopenia, the stress induced by occlusal disharmony appears to exert a more severe effect on bone metabolism, with both cortex-related and trabecular indices being significantly decreased. The reduction in the mechanical strength of long bones, which is known to be proportionally correlated to cortical bone-related parameters^22^, confirmed the severe changes in bone metabolism in the occlusal disharmony model.

Our histomorphometric analyses showed that occlusal disharmony-induced stress reduced bone mass in the long bones of the hind limbs of mice through a decrease in bone formation parameters. Specifically, serum levels of systemic metabolic markers were significantly lower in the disharmony group than in the control group (p < 0.0001), indicative of a decrease in bone formation parameters, but without an increase in bone resorption parameters (Figs 5–7), which was consistent with the findings of previous studies that have evaluated the effects of glucocorticoids in rodents^23–25^. Some previous studies, however, have reported an effect of glucocorticoids on bone resorption^26–28^. We examined bone resorption markers in the middle of the experimental period of observation. The serum level of TRACP 5b, which is indicative of bone resorption activity, did increase significantly in the disharmony group on post-induction day 5 (Supplementary Fig. S4b). We also examined the expression levels of *Rankl*, *Opg*, *Dkk1 and Sost* mRNA expression in the tibia and observed a trend to increasing levels in the *Rankl/Opg* mRNA expression ratio, with decreasing levels in the *Dkk1 and Sost* mRNA expression ratio, on post-induction day 5 (Supplementary Figs S6 and S7). Furthermore, histopathological examination of cortical bone in the femur was indicative of a high turnover rate of bone remodelling in the middle of the observation period (Supplementary Figs S3 and S4).

In conclusion, occlusal disharmony-induced stress could decrease bone formation and increase bone resorption, leading to osteopenia and reduced mechanical strength of bones, through its effect in increasing serum glucocorticoid levels. To our knowledge, this is the first report to have addressed the possible pathological effects of occlusal disharmony-induced stress on the bone mass and mechanical strength.

## Methods

### Animals

Five-week-old male C57BL/6J mice were obtained from Sankyo Labo Service Co., Ltd. (Tokyo, Japan). Mice were maintained in our animal care facility under room temperature (21 ± 1 °C), with a 12-h light/12-h dark cycle. The experimental procedures were reviewed and approved by the Institutional Animal Care and Use Committee of the Tokyo Medical and Dental University (Tokyo, Japan, approved number; #0130279A) and all experiments were performed in accordance with relevant guidelines and regulations. Since the mice in the occlusal disharmony group could not easily eat the standard (pellet) diet, the standard diet was changed to a powdered diet on the third day after the standard diet was provided. The mice had three- and six-day acclimation periods, respectively, to adapt to the powdered diet and single cages prior to the experimental intervention (day 0; Fig. 1).

Mice were randomly divided into two groups: a control group (n = 5) and an occlusal disharmony (disharmony) group (n = 5). In the disharmony group, the occlusal height was increased by 0.5 mm, using a composite resin (MI FIL, GC Co. Ltd., Tokyo, Japan), with the mice under anaesthesia, as per methods that have previously been described, but with some modifications^2,3^ (Fig. 1). The mice were subjected to anaesthesia using medetomidine hydrochloride (0.5 mg/kg; Domitor, Meijiseika, Tokyo, Japan) and ketamine hydrochloride (50 mg/kg; Ketalar, Sankyo, Tokyo, Japan). The same anaesthetic was used in mice in the control group, without any intervention. Atipamezole hydrochloride (the antagonist of medetomidine hydrochloride, 2.5 mg/kg; Nippon Zenyaku Kogyo Co., Ltd., Japan) was administered to all animals to reduce the stress from the anaesthesia^29^.

### Serum corticosterone measurements

The serum was separated from blood samples collected from the orbital vein under anaesthesia on days 1, 3 and day 7 after induction of occlusal disharmony. Each blood sampling procedure was completed within 30 s from the time of contact with a mouse. The separated serum sample was frozen at −80 °C until corticosterone measurements were performed. The serum corticosterone levels were determined using the Corticosterone HS EIA kit (Immunodiagnostic Systems Ltd., UK), according to the manufacturer’s instructions.

### Three-dimensional micro-CT analysis

After sacrifice, the tibiae, femurs and the third lumbar vertebrae were dissected, and the soft tissues were roughly removed. Bones were fixed in PBS-buffered glutaraldehyde (0.25%)-formalin (3.7%) solution (pH 7.4) for 2 days at 4 °C, and washed with PBS for radiological analysis.

A desktop X-ray micro-CT system (SMX-100CT, Shimadzu, Kyoto, Japan) was used to investigate the structural changes of the trabecular bone of the lumbar vertebra. The region of interest (ROI) for structural morphometry was the third lumbar vertebra, as shown in Fig. 3a. To separate trabecular bone from the bone marrow, the same threshold level was applied in the three-dimensional image analysis software program (TRI/3-D-BON, Ratoc System Engineering, Tokyo, Japan). The bone volume fraction was measured as the percentage of bone volume per tissue volume (BV/TV, %). Trabecular thickness (Tb.Th, μm), trabecular number (Tb.N, 1/mm), and the trabecular bone pattern factor (TBPf, 1/mm) were also measured.

### Bone mineral density measurements

The bone mineral density of the proximal metaphysis of the tibia and the distal- and mid-shaft regions of the femurs were measured by peripheral quantitative computed tomography (pQCT; XCT Research SA + , Stratec Medizintechnik GmbH, Pforzheim, Germany). We also evaluated the strength-strain index (SSI) and torsion index, which provide a measure of bone strength and strength under torsion, respectively^22^.

### Biochemical markers of bone formation/resorption-related parameters

Serum levels of tartrate-resistant acid phosphatase 5b (TRACP 5b) and osteocalcin were analysed using EIA kits (TRACP 5b; MBL CO., LTD, Nagoya-city, Japan, osteocalcin; Takara-Bio Inc, Otsu-city, Japan). The urine carboxy-terminal collagen crosslink (CTX) level was measured using Rat Laps (CTX-I) EIA kit (MBL CO., LTD, Nagoya-city, Japan), according to the manufacturers’ instructions. CTX levels were measured in both groups of mice, before and after occlusal disharmony induction.

### Histological assessment

Tibial bones were embedded in glycol methacrylate monomer (GMA). In brief, polymerization was performed at 4 °C. Standard un-decalcified sections (3 μm) were prepared using a fully automated rotary microtome Leica RM2255 device (Leica Biosystems, Nussloch GmbH, Germany). The direction of the cuts made in the bones was guided by μCT images of embedded samples before the sections were made. The sections were then stained with tartrate-resistant acid phosphatase (TRAP) and counterstained with toluidine blue. Histomorphometric analyses were performed using an image analysis system (KS400; Carl Zeiss, Jena, Germany), as per methods previously described^30,31^.

### Bone histomorphometry of the tibial metaphysis

To investigate secondary effects of occlusal disharmony on bone, a standard histomorphometric analysis of the tibial metaphysis was performed using the KS400 image analysing system^31–33^.

### Statistical analysis

The Kolmogorov–Smirnov test was used as a goodness of fit test to confirm the normal distribution of the data. Differences in measured outcomes between the control and disharmony group were evaluated using un-paired Student’s t-tests. A p-value < 0.05 was considered statistically significant.

## Electronic supplementary material

Supplementary information
