# Supplementary material for: Occlusal disharmony-induced stress causes osteopenia of the lumbar vertebrae and long bones in mice
Source: Sci Rep. 2018 Jan 9;8:173. doi: 10.1038/s41598-017-18037-y (PMC5760568; doi:10.1038/s41598-017-18037-y)
Supplement: Supplementary file 1 — Supplementary information [file 41598_2017_18037_MOESM1_ESM.pdf]

Title:

Occlusal disharmony-induced stress causes osteopenia of the lumbar vertebrae and long bones in mice

Authors:

Yasuhiro Shimizu[1], Masud Khan[2,3,4], Genki Kato[4], Kazuhiro Aoki[2]*, Takashi Ono[1]

[1] Department of Orthodontic Science, Graduate School of Medical and Dental Sciences, Tokyo Medical and Dental University (TMDU), 1-5-45 Yushima, Bunkyo-ku, Tokyo 113-8549, Japan

[2] Department of Basic Oral Health Engineering, Graduate School of Medical and Dental Sciences, Tokyo Medical and Dental University (TMDU), 1-5-45 Yushima, Bunkyo-ku, Tokyo 113-8549, Japan

[3] JSPS International Research Fellows, 1-5-45 Yushima, Bunkyo-ku, Tokyo 113-8549, Japan

[4] Department of Bio-Matrix (Pharmacology), Graduate School of Medical and Dental Sciences, Tokyo Medical and Dental University (TMDU), 1-5-45 Yushima, Bunkyo-ku, Tokyo 113-8549, Japan

* Corresponding author:

E-mail: kazu.hpha@tmd.ac.jp

Tel.: +81-3-5803-4641

Supplementary materials

Methods

Animals

Twenty-four, five-week-old male C57BL/6J mice were randomly divided into two groups: a control group (n=12) and an occlusal disharmony (disharmony) group (n=12). In the disharmony group, the occlusal height was increased by 0.5 mm, using a composite resin, with the mice under anaesthesia, as per the methods that have previously been described. The mice were subjected to anaesthesia using medetomidine hydrochloride (0.5 mg/kg; Domitor, Meijiseika, Tokyo, Japan) and ketamine hydrochloride (50 mg/kg; Ketalar, Sankyo, Tokyo, Japan). The same anaesthetic was used for the mice in the control group, without any intervention. Mice in each group were killed at days 1, 3 and 5 for examination of the time course of bone formation/resorption-related parameters.

Serum corticosterone measurements

The serum was separated from blood samples collected from the orbital vein under anaesthesia on day 3 after occlusal disharmony induction. The blood sampling procedure was completed within 30 s, from the time of contact with a mouse. The separated serum sample was frozen at −80 ºC until corticosterone measurements were performed. The serum corticosterone levels

were determined using the Corticosterone HS EIA kit (Immunodiagnostic Systems Ltd., UK), according to the manufacturer's instructions.

Biochemical markers of bone formation/resorption-related parameters

Serum levels of tartrate-resistant acid phosphatase 5b (TRACP 5b) and osteocalcin on days 3 and 5 were analysed using EIA kits (TRACP 5b; MBL CO., LTD, Nagoya-city, Japan, osteocalcin; Takara-Bio Inc, Otsu-city, Japan).

Histological assessment of the cortical bone area of the femur on day 7

Un-decalcified frozen transverse sections, 5 μm in thickness, were prepared, as described elsewhere[1]. The mineralizing surface (MS) and bone volume (BV) were measured using a KS400 image analysing system, as previously described[2, 3].

Histological examination of osteocyte apoptosis in the cortical bone area

To investigate the increase in osteocyte apoptosis in the cortical bone area, un-decalcified frozen sections obtained for histological assessment of the femur on day 3 were stained with DNA-specific fluorescent Hoechst 33258.

mRNA analyses

Quantitative RT-PCR analysis was performed using PCR Thermal Cycler Dice (Takara, Kyoto, Japan), according to the manufacturer's instructions. The following mouse-specific primers were

used: *mGapdh forward; TGC ACC ACC AAC TGC TAA G, mGapdh reverse; GGA TGC AGG GAT GAT GTT C, mSost forward: GAG AAC AAC CAG ACC ATG AAC, mSost reverse: GCT CGC GGC AGC TGT ACT, mDkk1 forward: GAA TAT GCA TGC CCT CTG AC, mDkk1 reverse: TCA GTG TGG TTC TTC TGG GA, Rankl forward: ACC AGC ATC AAA ATC CCA AG, Rankl reverse: TTG AAA GCC CCA AAG TAC GT, Opg forward: GCC GAG AGT GTA GAG AGG, Opg reverse: GTT CGA GTG GCC GAG ATG.* The relative levels of expression were calculated by the ΔΔCt method.

Statistical analysis

Differences in the measured outcomes between the control and disharmony group were evaluated using an unpaired Mann–Whitney *U*-test. Multiple intergroup comparisons were performed using a one-way analysis of variance (ANOVA). When a significant F-ratio was identified, between-group comparisons were performed using Fisher's protected least significant difference (PLSD) as a post-hoc test. A P-value < 0.05 was considered statistically significant.

Supplementary References

1. Suzuki, N. et al. A threshold of mechanical strain intensity for the direct activation of osteoblast function exists in a murine maxilla loading model. *Biomech Model Mechanobiol.*

15, 1091-1100, (2016).

2. Kato, G. et al. The inhibitory effects of a RANKL-binding peptide on articular and periarticular bone loss in a murine model of collagen-induced arthritis: a bone histomorphometric study. *Arthritis Res Ther*. **17**, 251 (2015).

3. Dempster, D.W. et al. Standardized nomenclature, symbols, and units for bone histomorphometry: a 2012 update of the report of the ASBMR Histomorphometry Nomenclature Committee. *J Bone Miner Res*. **28**, 2-17 (2013).

Supplementary Figure legends

Supplementary Figure S1. Measurements of serum corticosterone levels on day 3 after induction of occlusal disharmony. Values are expressed as the mean±standard deviation; Ctr, control group; Dis, occlusal disharmony group; *, $p < 0.05$ versus the control group (Ctr).

Supplementary Figure S2. A representative pQCT image of the femur for quantitative analyses (bar, 1 mm) is shown in (a). The colour calibration for bone mineral density is shown in the right bar. The reduction in the maximum bone mineral density with occlusal disharmony is indicated. Other measured variables of bone structure are shown as follows: total mineral density of the femur (b); total mineral density (c); cortical bone area (d); cortical thickness (e); trabecular

density (f); strength strain index (SSI), (g) torsion index. Values are expressed as the mean±standard deviation; Ctr, control group; Dis, occlusal disharmony group; *, $p < 0.05$; **, $p < 0.01$, ***, $p < 0.001$ versus the control group (Ctr).

Supplementary Figure S3. A diagram with the square showing the area of the region of fluorescence images in the transverse sections of the femur (top row). Fluorescence images of un-decalcified frozen transverse sections of the right side of the femur on day 5 are shown (bottom row, bar, 100 µm). Ctr, control group; Dis, occlusal disharmony group.

Supplementary Figure S4. Bone formation/resorption-related parameters. (a) Bone formation parameter (MS/BV) of the cortical bone of the femur, with calcein injections administered at the beginning of occlusal disharmony (day 0) and on post-induction day 6. (b) Apoptotic cortical bone cells; experimental versus control on day 3, bone resorption parameters of the serum TRACP 5b level on day 5, and the number of osteoclasts (N.Oc) in the intra-cortical region on day 5. Values are expressed as the mean±standard deviation; Ctr, control group; Dis, occlusal disharmony group; *, $p < 0.05$ versus the control group (Ctr).

Supplementary Figure S5. Multiple intergroup comparisons of measured serum corticosterone levels on days 1, 3, and 7 after induction of occlusal disharmony. Values are expressed as the mean±standard deviation; Ctr 1, control group on day 1; Dis 1, disharmony group on day 1; Ctr

3, control group on day 3; Dis 3, disharmony group on day 3; Ctr 7, control group on day 7; Dis 7, disharmony group on day 7; *, $p < 0.05$ versus the disharmony group (Dis) on day 3 and $^{\#}$, $p < 0.05$ versus Dis on day 1. ^{a}, ^{b} and ^{c}, $p < 0.05$ versus the control group (Ctr) on days 1, 3, and 7, respectively.

Supplementary Figure S6. Gene expression analyses of *Rankl* (top row) and *Opg* (middle row) in the whole tibiae by quantitative PCR on days 1, 3, and 5. *Rankl/Opg* ratios are presented in the bottom row. Values are expressed as mean±standard deviation; Ctr, control group; Dis, occlusal disharmony group; *, $p < 0.05$ versus the control group (Ctr).

Supplementary Figure S7. Gene expression analyses of *Dkk1* (top row) and *Sost* (bottom row) in the tibiae by quantitative PCR on days 1, 3, and 5. A similar tendency between *Dkk1* expressions and *Sost* expressions on days 1, 3, and 5 was apparent, although these differences were not significant. Values are expressed as the mean±standard deviation; Ctr, control group; Dis, occlusal disharmony group.

Supplementary Figure S8. An illustrated summary of the experimental results. In the initial stage after induction of occlusal disharmony, bone formation parameters were suppressed (Fig. 5e). However, the suppression of bone formation markers on days 1 and 3 was no longer evident on day 7 (Fig. 5f) and was accompanied by a decrease in the rate of increase in the serum level

of corticosterone in the disharmony group (Fig. 2, Supplementary Figs. S1 and S5). In terms of the bone resorption parameters, apoptotic cortical bone cells on post-induction day 3 were significantly increased, which might be the reason for the increase in the bone resorption marker levels on day 5 (Supplementary Fig. S4b). Histomorphometric analyses indicate a high turnover rate of bone remodelling in the middle of the observation period (Supplementary Figs. S3 and S4).

BR: bone resorption, BF: bone formation, red arrow: significant difference

Supplementary Fig. S1

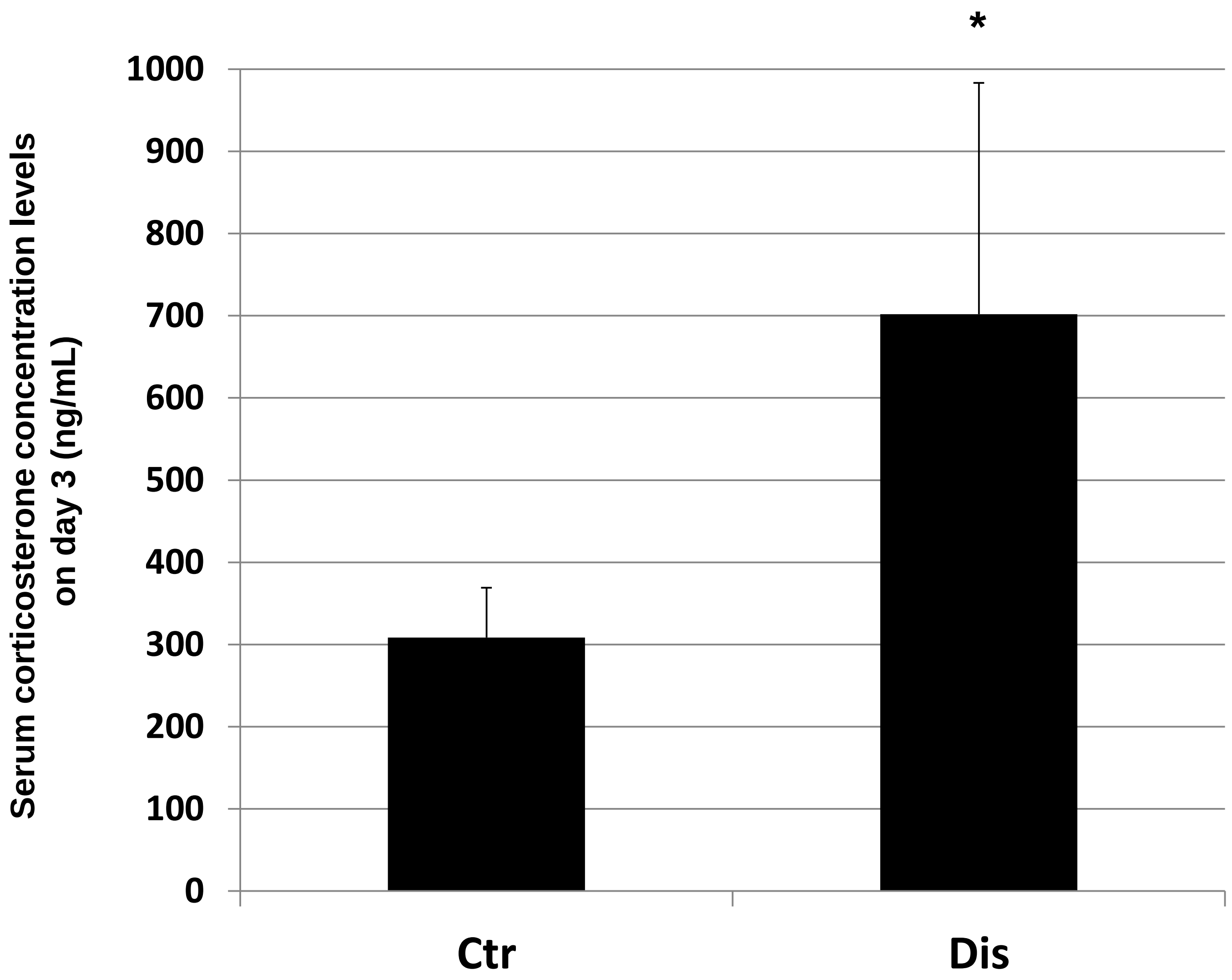

Supplementary Fig. S2

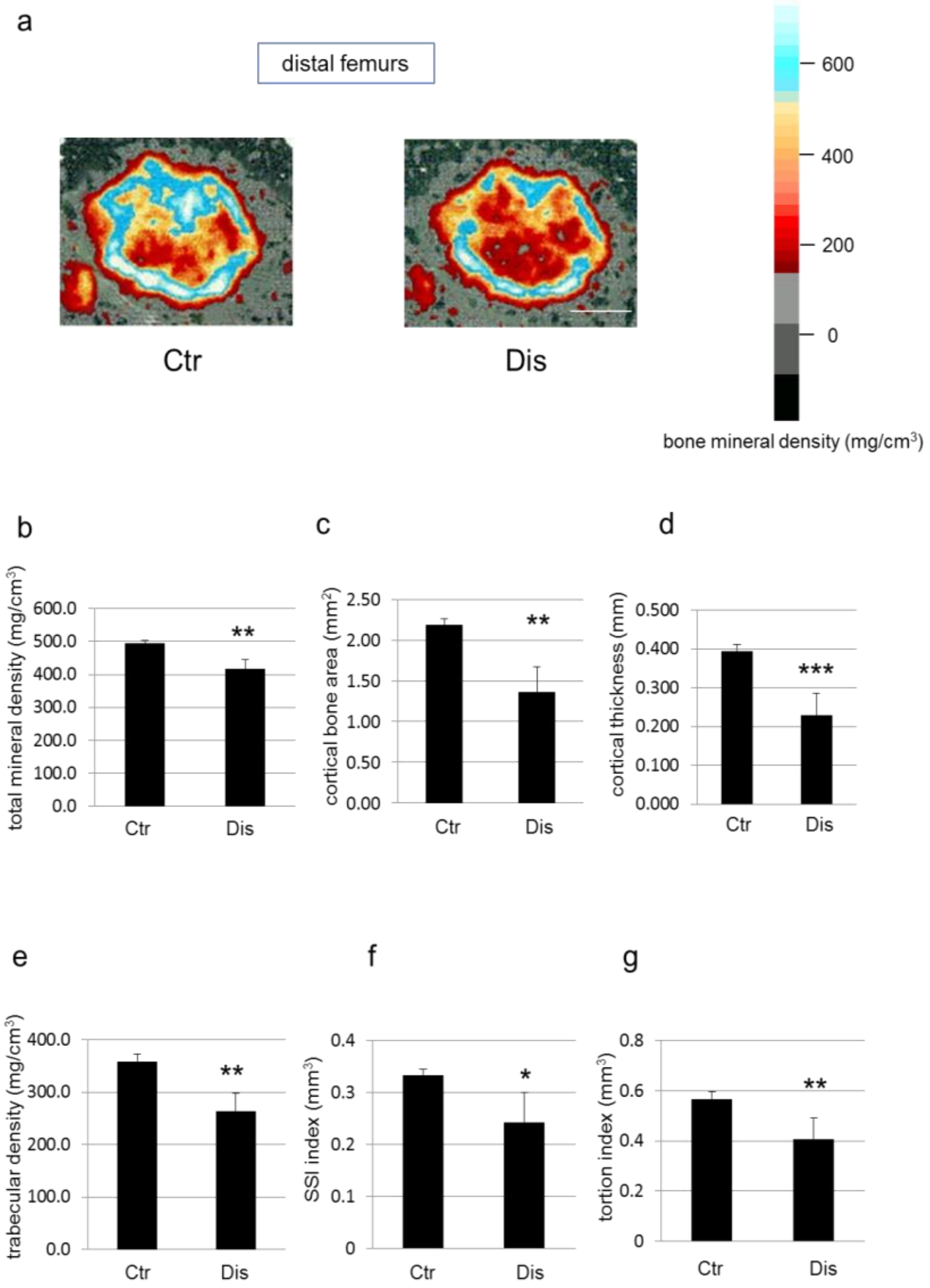

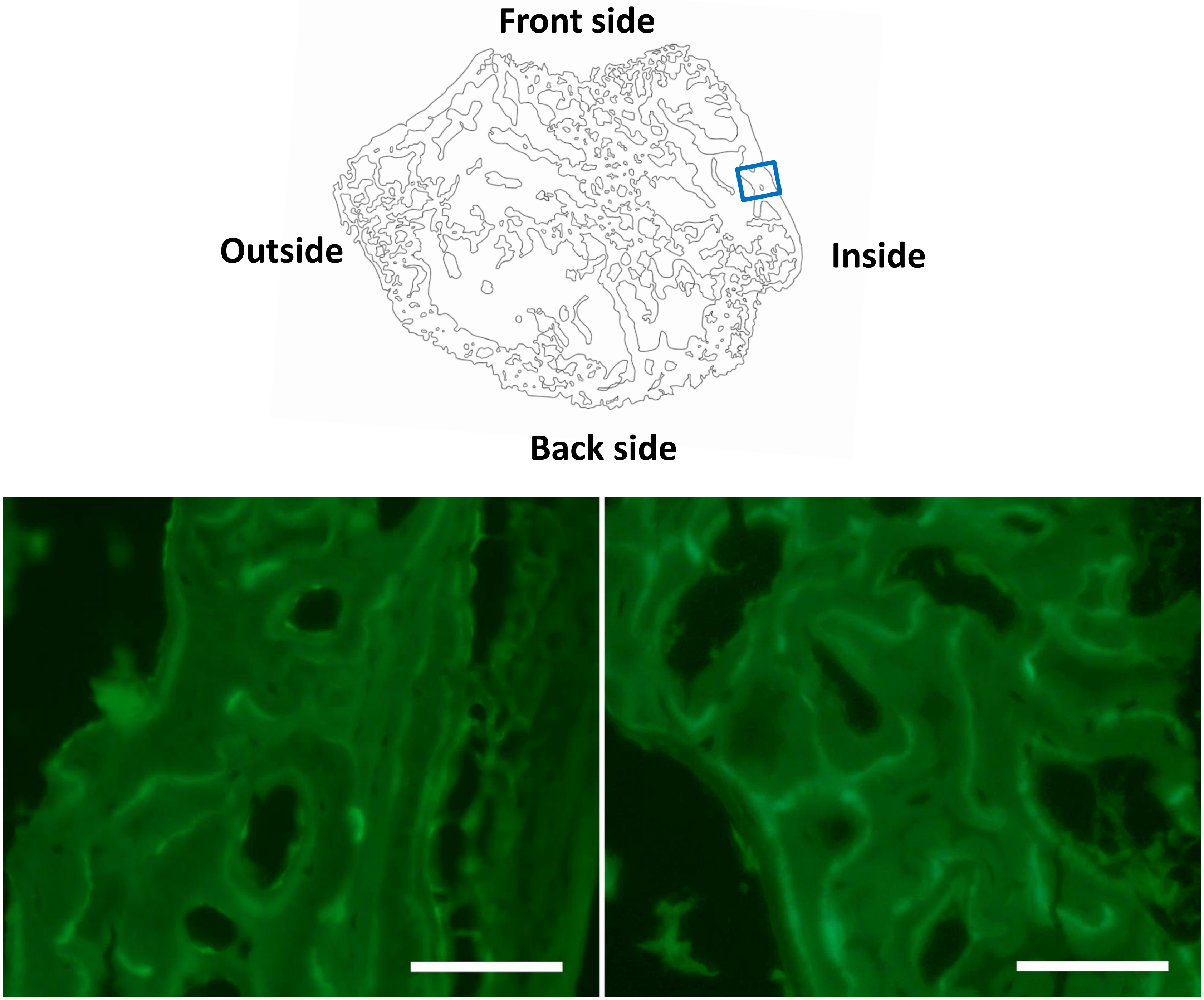
Front side
Outside
Inside
Back side

Supplementary Fig. S4

a

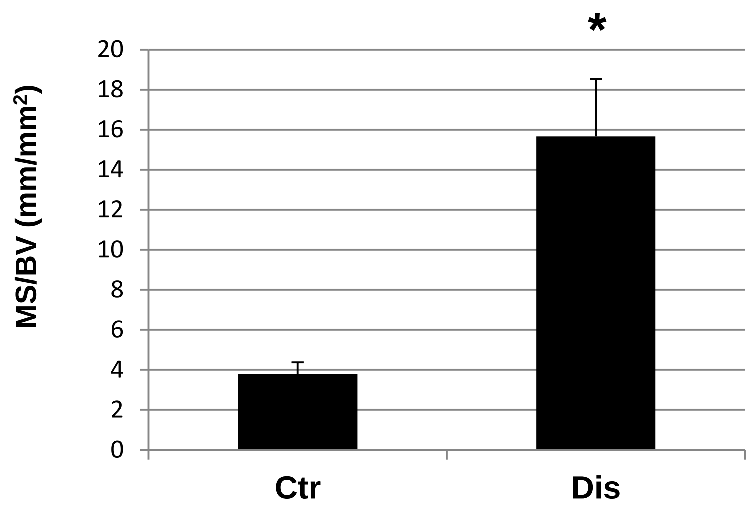

b

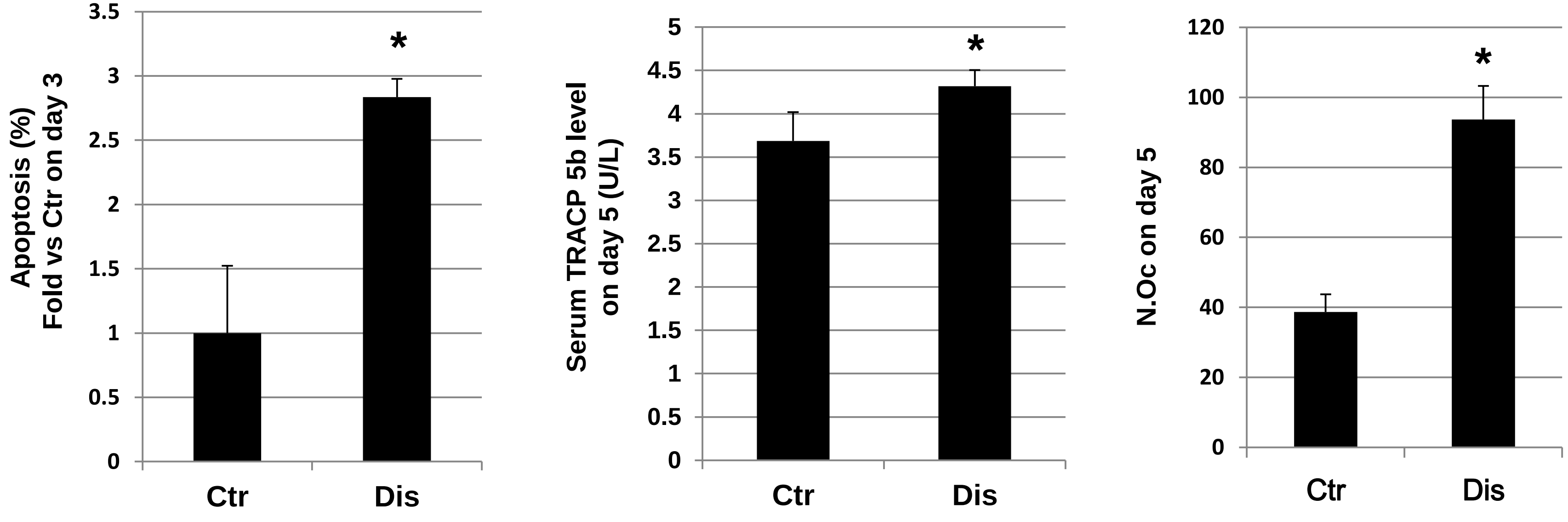

Supplementary Fig. S5

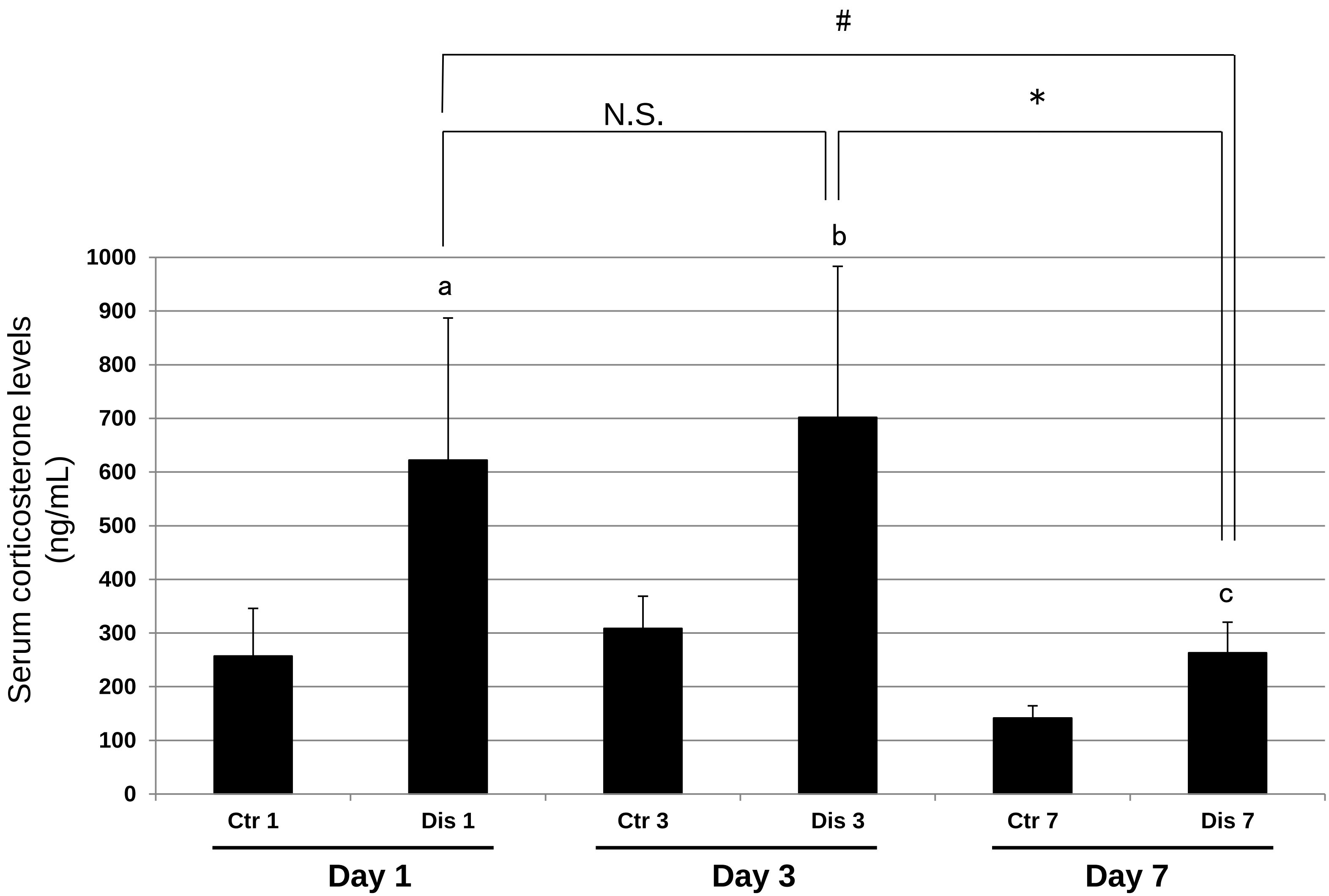

Supplementary Fig. S6

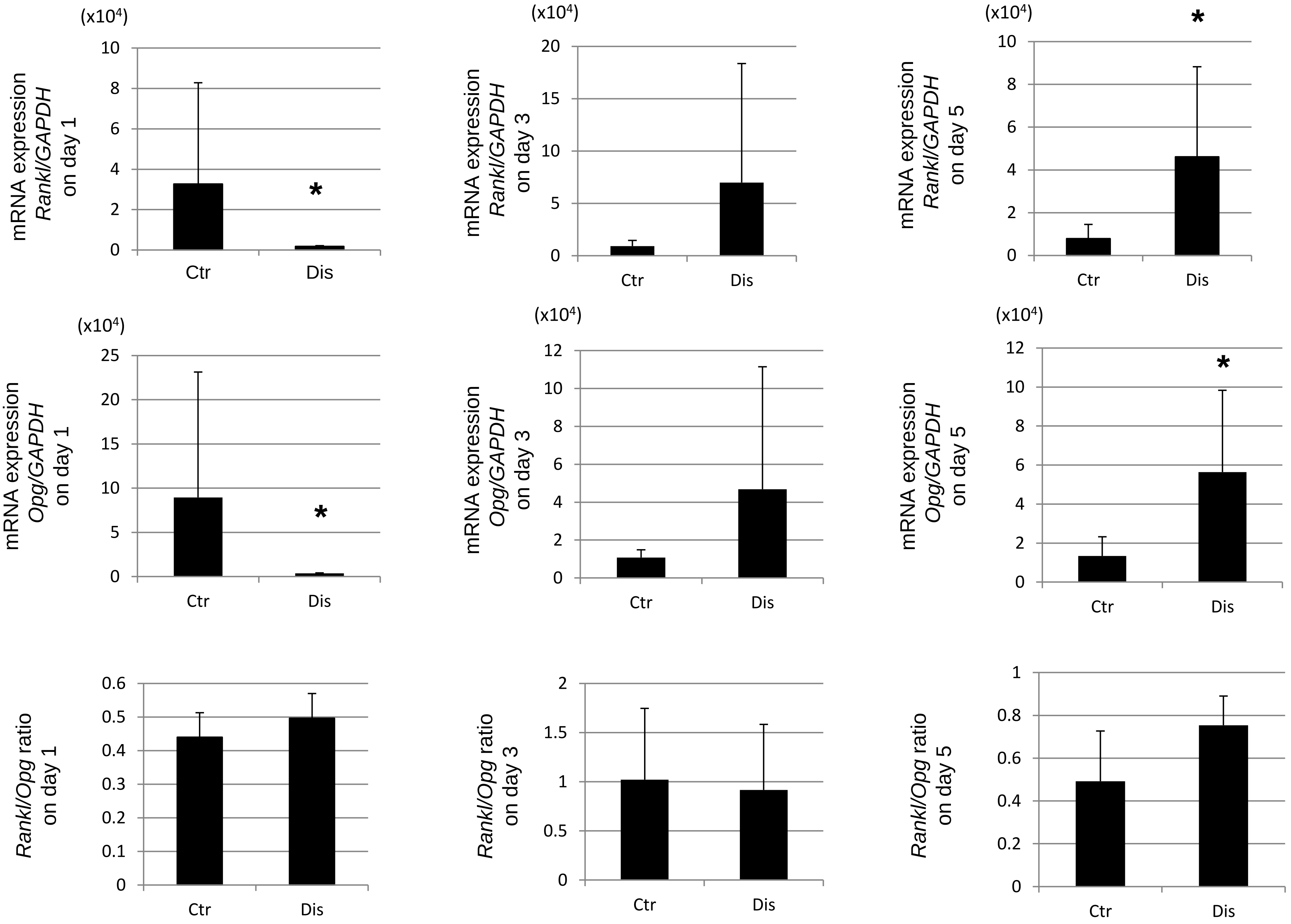

Supplementary Fig. S7

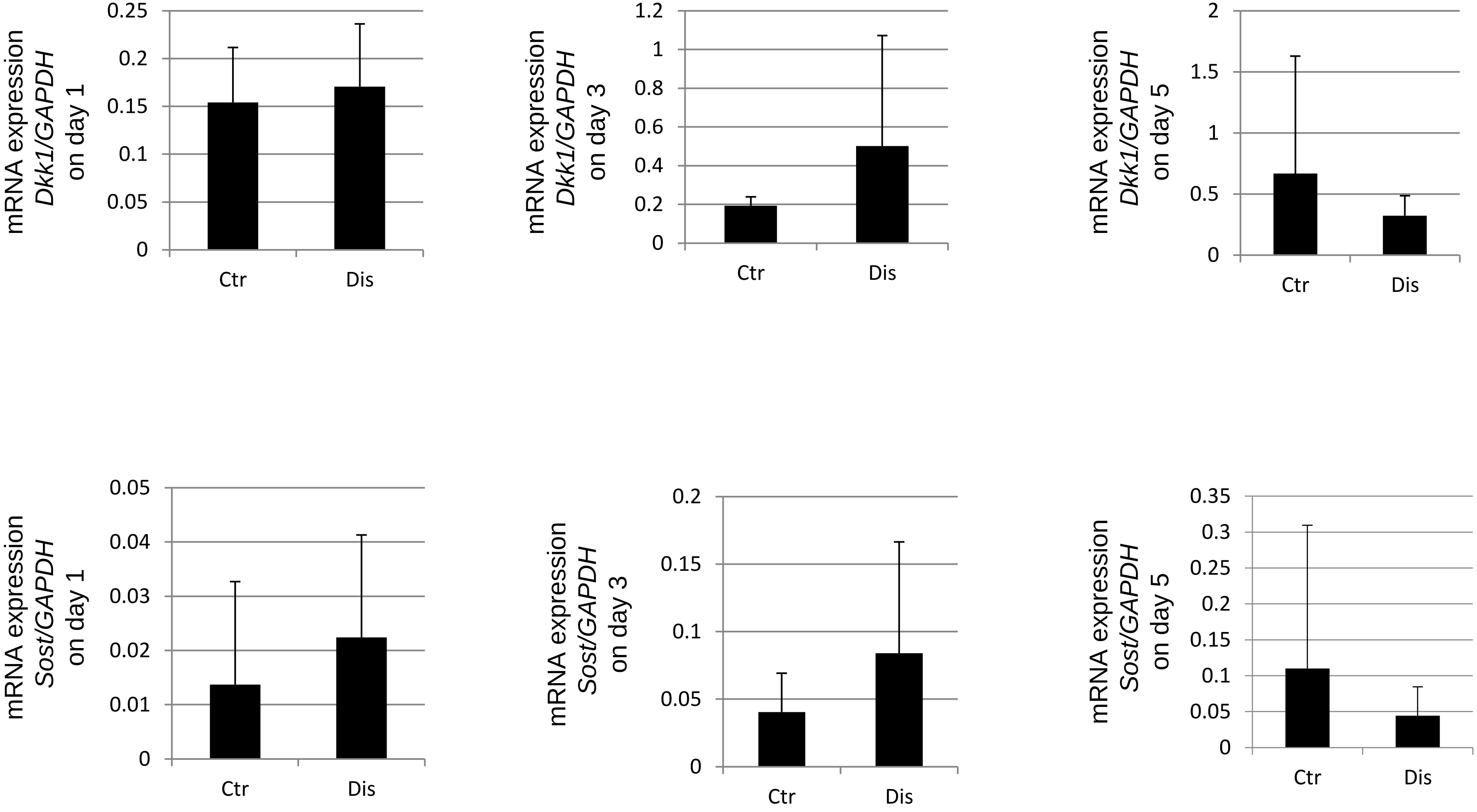

Supplementary Fig. S8

Days after the induction of occlusal disharmony		Day 1	Day 3	Day 5	Day 7
**Serum marker**	**BR**(TRACP 5b)	→	→	↑	↓
	**BF**(osteocalcin)	↓↓↓	↓	↘	→
**Gene**	**BR** (*Rankl/Opg*)	→	→	↗	
	**BF** (1/*Dkk1*,1/*Sost)*	↘	↘	↗	
**Remodeling of cortical bone**	**BR**(Apoptotic cortical bone cells, N.Oc)		↑	↑	
	**BF** (MS/BV)				↑
